# Biomechanical evaluation of 3D-printed porous lattice versus solid mandibular implants: an in vitro study

**DOI:** 10.1038/s41598-026-50741-6

**Published:** 2026-04-27

**Authors:** Hao Zhang, Jui-Ting Hsu, Lih-Jyh Fuh, Heng-Li Huang

**Affiliations:** 1 School of Dentistry, China Medical University, Taichung, Taiwan; 2 Department of Biomedical Engineering, China Medical University, Taichung, Taiwan; 3 Department of Dentistry, China Medical University Hospital, Taichung, Taiwan; 4 Department of Bioinformatics and Medical Engineering, Asia University, Taichung, Taiwan

**Keywords:** Mandibular reconstruction, Porous implant, Stress shielding, Additive manufacturing, Strain gauge, Biotechnology, Engineering, Health care, Materials science, Medical research

## Abstract

Segmental mandibular reconstruction is challenging. This in-vitro study compared the biomechanics of two 3D-printed porous Ti-6Al-4 V implants (Quad-diametral-cross, Hex-vase) versus a solid implant using a dual-material 3D-printed mandibular model and strain gauges. A 100 N static load was applied. Under eccentric loading, the solid implant generated extremely high tensile strain (871.67 µε), while porous designs reduced peak strains. The Hex-vase design transmitted the highest compressive strain (-795.85 µε), 65% greater in magnitude than the solid, suggesting improved mechanical stimulus transfer to peri-implant bone. The QDC design provided a moderate environment, suitable for compromised bone. Porous implants are biomechanically superior, offering customizable solutions to mitigate stress shielding.

## Introduction

Segmental mandibular defects present a significant challenge in oral and maxillofacial surgery^1^. If not properly reconstructed, these defects can severely impair fundamental physiological functions, such as mastication and speech, and lead to alterations in facial appearance, profoundly impacting the patient’s psychological and social well-being^2^. Currently, the fibular free flap remains the gold standard for mandibular reconstruction^3–5^. However, this surgical procedure is not only lengthy and complex but is also associated with risks of donor site morbidity^6,7^. Furthermore, the harvested bone segment requires manual contouring by the surgeon based on clinical experience, making it difficult to accurately reproduce the precise anatomical morphology of the mandible.

The advancement of additive manufacturing has introduced breakthroughs in craniofacial reconstruction through patient-specific medical implants^8,9^. Titanium alloys have become the material of choice for orthopedic implants owing to their excellent biocompatibility, corrosion resistance, and high strength-to-weight ratio^10,11^. 3D printing technology enables the fabrication of not only solid implants that precisely fit the patient’s defect but also designs incorporating porous lattice structures. These structures significantly reduce the implant’s weight, and their interconnected pores are designed to guide the ingrowth of surrounding bone tissue, thereby facilitating osseointegration^12,13^. Furthermore, by tailoring the parameters of the porous lattice structure, the porous lattice designs reduced the effective modulus by up to 98% compared to solid Ti-6Al-4 V, allowing approximation to trabecular bone stiffness. This mitigates the stress shielding effect and prevents bone resorption around the implant, which is often caused by insufficient mechanical stimulation^14–16^.

In our previous study^17^, comprehensive biomechanical simulations of four distinct porous lattice designs—Tetrahedron, Hex-star, Hex-vase, and Quad-diametral-cross—were conducted using the Finite Element Analysis (FEA) method. The findings indicated that the Hex-vase and Quad-diametral-cross (QDC) designs exhibited superior potential in terms of stress distribution, effectively dispersing stress and preventing excessive stress concentration. However, FEA is ultimately a computational simulation, and its results are influenced by idealized assumptions such as material property definitions, boundary conditions, and loading configurations. Therefore, the predicted mechanical behavior still requires final validation through physical experimentation^18^.

Therefore, the primary objective of this study is to bridge the findings of the preceding computational simulations with in-vitro experimental validation. Mandibular implants featuring the ‘Hex-vase’ and ‘QDC’ porous designs were fabricated using 3D printing technology, with a solid implant serving as the control group. By affixing strain gauges to a mandible model reconstructed from a patient’s CBCT images and applying a static load, this research aims to: (1) directly measure and compare the strain distribution on the peri-implant bone for the two porous lattice designs; and (2) empirically determine whether these porous designs are superior to the solid design in effectively transferring load to the bone, thereby supporting their theoretical advantage in mitigating stress shielding.

## Results

Tables 1 and 2 present the maximum and minimum principal strains measured at four locations (L1, L2, R1, and R2) of the bone model for the three implant designs—Quad-diametral-cross (QDC), Hex-vase, and Solid—under an axial load of 100 N during the in-vitro experiments.

**Table 1:** Maximum and minimum principal strains and statistical comparisons under axial loading at Point A (unit: µstrain).

Force A		Quad-diametral-cross	Hex-vase	Solid	*P*-value
Maximum Principal strain (Tensile strain)	L1	787.32 ± 6.64^aA^	779.98 ± 11.02^aA^	573.30 ± 21.51^bA^	< 0.0001*
	L2	441.40 ± 13.00^aB^	664.66 ± 21.95^bB^	420.07 ± 11.32^aB^	< 0.0001*
	R1	237.26 ± 3.33^aC^	370.97 ± 12.54^bC^	264.23 ± 16.42^cC^	< 0.0001*
	R2	205.42 ± 5.41^aD^	288.19 ± 9.76^bD^	250.23 ± 14.28^abC^	< 0.0001*
	P-value	< 0.0001*	< 0.0001*	< 0.0001*	
Minimum Principal strain (Compressive strain)	L1	−392.04 ± 8.94^aA^	−218.69 ± 7.01^bA^	−363.44 ± 8.22^abA^	< 0.0001*
	L2	−224.54 ± 5.89^aB^	−777.81 ± 10.20^bB^	−337.07 ± 18.02^cB^	< 0.0001*
	R1	−213.54 ± 4.02^aC^	−192.26 ± 6.33^abC^	−115.95 ± 9.08^bC^	< 0.0001*
	R2	−16.28 ± 3.53^aD^	−207.47 ± 8.06^bA^	−158.80 ± 8.11^cD^	< 0.0001*
	P-value	< 0.0001*	< 0.0001*	< 0.0001*	

1. P-values were calculated using one-way ANOVA for normally distributed data, and the Kruskal–Wallis test for non-normally distributed data; post hoc comparisons were performed using Bonferroni and Tukey tests for ANOVA, and Dunn–Bonferroni for Kruskal–Wallis.

2. Different lowercase letters (a, b, c) indicate statistically significant differences among different implant designs at the same measurement location, whereas different uppercase letters (A, B, C, D) indicate statistically significant differences among different measurement locations within the same implant design (*p* < 0.05).

3. * indicates statistical significance at α = 0.05.

**Table 2:** Maximum and minimum principal strains and statistical comparisons under axial loading at Point B (unit: µstrain).

Force B		Quad-diametral-cross	Hex-vase	Solid	*P*-value
Maximum Principal strain (Tensile strain)	L1	551.40 ± 3.95^aA^	630.94 ± 3.79^bA^	356.39 ± 21.44^cA^	< 0.0001*
	L2	295.00 ± 4.09^aB^	517.27 ± 17.79^bB^	239.60 ± 16.41^cB^	< 0.0001*
	R1	350.80 ± 6.16^aC^	553.39 ± 11.70^bC^	871.67 ± 55.94^cC^	< 0.0001*
	R2	363.40 ± 13.44^aD^	404.20 ± 9.24^bD^	706.01 ± 27.68^cD^	< 0.0001*
	P-value	< 0.0001*	< 0.0001*	< 0.0001*	
Minimum Principal strain (Compressive strain)	L1	−301.26 ± 4.27^aA^	−205.37 ± 3.68^bA^	−174.10 ± 13.44^cA^	< 0.0001*
	L2	−147.28 ± 2.76^aB^	−795.85 ± 12.76^bB^	−250.46 ± 16.86^abB^	< 0.0001*
	R1	−184.80 ± 2.61^aC^	−268.25 ± 5.08^bC^	−482.38 ± 35.90^cC^	< 0.0001*
	R2	−84.40 ± 2.78^aD^	−287.78 ± 6.89^bD^	−449.15 ± 36.13^cC^	< 0.0001*
	P-value	< 0.0001*	< 0.0001*	< 0.0001*	

1. P-values were calculated using one-way ANOVA for normally distributed data, and the Kruskal–Wallis test for non-normally distributed data; post hoc comparisons were performed using Bonferroni and Tukey tests for ANOVA, and Dunn–Bonferroni for Kruskal–Wallis.

2. Different lowercase letters (a, b, c) indicate statistically significant differences among different implant designs at the same measurement location, whereas different uppercase letters (A, B, C, D) indicate statistically significant differences among different measurement locations within the same implant design (*p* < 0.05).

3. * indicates statistical significance at α = 0.05.

### Effect of loading position on strain distribution for different implant designs

Under the load applied at Point A, the resulting strain for all designs was predominantly concentrated on the left side of the model (L1 and L2). Specifically, the QDC implant induced the highest tensile strain at the L1 location (787.32 ± 6.64 µε), whereas the Hex-vase implant generated the highest compressive strain at the L2 location (−777.81 ± 10.20 µε). The strain values recorded for the Solid implant under this loading condition were comparatively lower (Table 1).

When the loading point was shifted to Point B, the strain distribution changed significantly. The Solid implant induced the highest tensile strain across all tests (871.67 ± 55.94 µε) at the R1 location, indicating a notable strain concentration. Meanwhile, the Hex-vase implant continued to generate the highest compressive strain at the L2 location, with the value increasing to −795.85 ± 12.76 µε. The overall strain values for the QDC implant under this loading condition were comparatively low (Table 2).

A comprehensive comparison between the left and right sides reveals a distinct asymmetry in strain distribution. Under the load at Point A, all designs exhibited higher strain values on the left side (L1, L2). However, under the load at Point B, the tensile strain on the right side (R1, R2) increased significantly, particularly for the Solid implant. For this implant, not only was the highest tensile strain (871.67 µε) observed at the R1 location, but its overall tensile strains on the right side (R1: 871.67 µε, R2: 706.01 µε) were also substantially higher than those on the left side (L1: 356.39 µε, L2: 239.60 µε), demonstrating extreme strain concentration and asymmetry. This finding confirms that the occlusal loading point is a critical factor in modulating the strain balance between the bilateral sides of the bone, an effect that was most pronounced with the more rigid Solid implant.

### Tensile strain analysis

Among the three designs, the Solid implant generated the highest peak tensile strain, recording 871.67 µε at the R1 location when loaded at Point B. In contrast, the two porous designs exhibited lower peak values. The maximum tensile strain for the Quad-diametral-cross implant was 787.32 µε, occurring at the L1 location under the load at Point A, while the maximum value for the Hex-vase implant was 779.98 µε, observed at the same location and under the same loading condition.

Numerically, the peak tensile strain induced on the bone by the Solid implant was approximately 10.7% higher than that from the Quad-diametral-cross implant and 11.8% higher than that from the Hex-vase implant, respectively. This highlights the advantage of porous structures in dispersing tensile stress. Furthermore, the standard deviation (SD) associated with the peak strain measurement for the Solid implant was 55.94 µε, a value substantially greater than the SDs for the two porous designs (6.64 µε and 11.02 µε, respectively). This indicates that the mechanical response of the bone surrounding the solid implant exhibits lower stability.

### Compressive strain analysis

In terms of compressive strain, the Hex-vase design registered a peak value of −795.85 µε at the L2 location under the load at Point B. The magnitude of this strain was 65% greater than that of the Solid implant (−482.38 µε) and 103% greater than that of the Quad-diametral-cross implant (−392.04 µε). This suggests that the Hex-vase design may possess a more effective load transfer mechanism, capable of transmitting a higher magnitude of compressive strain to the bone.

## Discussion

Through in-vitro strain gauge experiments, this study validated and compared the biomechanical performance of two porous lattice implants (Quad-diametral-cross and Hex-vase) against a conventional solid implant. A notable aspect of our methodology was the use of advanced, multi-material 3D printing with dual photopolymer resins (VeroWhitePlus and a Rubber-Like Digital Material) to simulate both cortical and cancellous bone. The results revealed that the porous implant designs facilitate a more effective transfer of occlusal loads to the surrounding bone, thereby potentially mitigating the risk of stress shielding^19–22^.

This study demonstrates that minor alterations in the loading point can significantly impact the overall strain distribution. From a structural mechanics perspective, eccentric loading increases the bending moment. The solid implant, due to its higher stiffness, asymmetrically increases moment transfer. In contrast, the porous implants allow slight compliance, resulting in better stress redistribution. As shown in the results, when the loading point was shifted from Point A to Point B, the region of strain concentration transferred accordingly. From a clinical perspective, this highlights the critical importance of the occlusal design and adjustment of the prosthesis^23,24^. Precise occlusal control, which ensures that forces are distributed evenly across the surrounding bone, is therefore an indispensable element for the long-term postoperative success of dental implants.

Beyond stress distribution, the ability of an implant to transmit physiological loading stimuli to the adjacent bone is also critical for long-term stability. This study found that the Hex-vase porous implant generated a higher magnitude of compressive strain (−795.85 µε) than the other designs. The observed compressive strain magnitude falls within the proposed physiological window of Frost’s mechanostat theory, suggesting potential mechanical favorability^25^.

The surface strain magnitude at measurement sites suggests differences in mechanical stimulus transmission, with the Hex-vase design implying greater mechanical stimulation to the surrounding bone. This is a crucial advantage because stress shielding is known to cause peri-implant bone resorption, which can ultimately compromise the long-term stability of the implant^26^. Therefore, this particular attribute of the Hex-vase design is beneficial, as it may mitigate the stress shielding effects caused by excessive implant rigidity^27^.

Although both porous implants demonstrated biomechanical advantages, specifically, lower tensile peak concentration, a more symmetric strain distribution, and reduced strain variability, over the solid design, they exhibited distinct mechanical characteristics, suggesting their suitability for different clinical applications. The Quad-diametral-cross (QDC) design exhibited a lower compressive strain magnitude compared with the Hex-vase design. Furthermore, its peak tensile strain was comparable to that of the Hex-vase implant and substantially lower than that of the solid design. These findings suggest that the QDC design tends to create a more moderate and balanced mechanical environment, which may represent a preferable option for patients with compromised bone quality or in cases where preservation of the remaining bone stock is critical.

Conversely, the Hex-vase design’s ability to generate higher compressive strain (−795.85 µε) on the bone suggests an enhanced potential for mechanical stimulus transfer to the peri-implant bone. This suggests it may be more suitable for treatment plans in patients with good bone quality who desire an enhanced healing response. While this study has not exhaustively explored the diverse mechanical behaviors of various porous implant designs, our preliminary analysis indicates that for the future design of patient-specific implants, clinicians should select a porous architecture based on the individual patient’s bone quality. Such a tailored approach is crucial for achieving more predictable and optimal therapeutic outcomes.

The limitations of this study are as follows. The mechanical boundary conditions and material properties in the present in-vitro study differ from those of our previous FEA simulation (e.g., synthetic resins vs. ideal human bone, and static vs. muscle-driven loading). Therefore, a direct quantitative correlation of absolute strain values is not feasible; however, the highly consistent qualitative trends successfully serve as a physical validation of the computational models. The elastic modulus of the material used to simulate cortical bone in this experiment (2–3 GPa) is slightly lower than the values reported in other studies^17,28,29^. This limits the direct applicability of the data from this in-vitro study for definitive clinical recommendations. Furthermore, the manufacturer’s technical data sheet for the rubber-like material used to simulate cancellous bone did not provide a key value for its elastic modulus. This absence of data restricted a complete and precise analysis of its mechanical response. Furthermore, the present study utilized a simplified 100 N static vertical load, which does not fully replicate the complex, dynamic, and multi-vectorial forces encountered during actual mastication. Future studies incorporating dynamic cyclic loading or varying force vectors are necessary to fully elucidate the long-term fatigue behavior and the multi-directional strain advantages of the porous designs.

In conclusion, under the experimental configuration and limitations, the following findings were obtained: Through in-vitro strain gauge experiments, this study validated and compared the biomechanical effects of two porous implants (Quad-diametral-cross and Hex-vase) and a conventional solid implant on the surrounding bone within a mandibular reconstruction model. The findings indicate that porous implants exhibit more advantageous biomechanical characteristics than their solid counterparts. Under eccentric loading, the solid implant failed to effectively distribute stress, instead transferring forces in a highly concentrated manner to the ipsilateral fixation points, resulting in extremely high localized tensile strain concentrations in the surrounding bone. In contrast, both porous designs effectively distributed stress and reduced the peak strains exerted on the bone. Furthermore, this study revealed that different porous implant designs can inform distinct clinical treatment strategies; for example, the Hex-vase design excelled at transmitting bone-stimulating compressive strains, whereas the lower peak strains of the Quad-diametral-cross design may render it a more suitable choice for patients with compromised bone quality.

## Methods

### Implant design and fabrication

The experimental protocol was conducted according to the guidelines of the Declaration of Helsinki and approved by the Institutional Review Board of China Medical University Hospital (Approval No. CMUH110-REC2-247). Cone-beam computed tomography (CBCT) images of the mandible were retrospectively obtained from a patient with a right-sided malignant tumor. Because the patient had completed treatment more than one year prior and was not scheduled for further postoperative follow-up, the need for informed consent was formally waived by the Institutional Review Board of China Medical University Hospital.

**Fig. 1:**
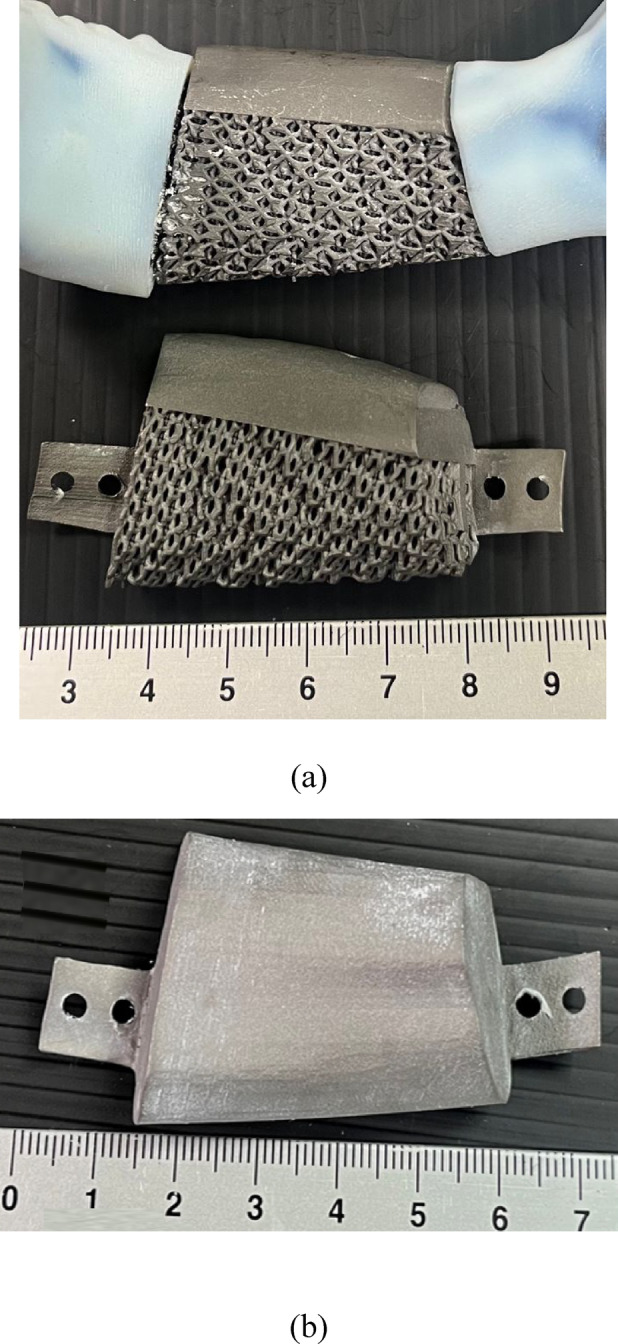
**a** The mandibular implants with porous lattice designs of quad-diametral-cross (upper) and hex-vase (lower). **b** The solid design of mandibular implant.

Two types of porous mandibular reconstruction implants—featuring Hex-vase (Fig. 1a) and Quad-diametral-cross (QDC) (Fig. 1a) internal architectures—were designed as the experimental groups, while an implant with a solid, non-porous internal structure served as the control group (Solid) (Fig. 1b). The external geometries of all implants were derived from patient-specific CBCT data obtained in our previous research. The three-dimensional patient-specific models were reconstructed using medical imaging software (Mimics, Version 14.0; Materialise, Belgium) and further refined using computer-aided design (CAD) software (SolidWorks, Version 2024; Dassault Systèmes, USA). Additionally, two Kerator Overdenture Attachment Systems (Kerator P&D, Seoul, South Korea) were incorporated into the superior region of each implant design to facilitate future prosthetic connection and restore the patient’s occlusal function. Corresponding cavities were pre-designed in the CAD models for the attachments. The Kerator attachments were not directly 3D printed; instead, they were secured into the printed titanium implants using an adhesive, without the application of preload torque.

All implants were fabricated using Ti-6Al-4 V powder with a particle size range of 15–53 μm and a density of 2.5 g/cm³. Manufacturing was performed with a Shining 3D EP M250 selective laser melting (SLM) system (Shining 3D Tech Co., Ltd., Hangzhou, China), equipped with a 500 W fiber laser featuring a spot size of 70 μm. The layer thickness was set to 20 μm, and the printing process was conducted in an argon-purged chamber with oxygen concentration maintained below 100 ppm to minimize oxidation. Upon completion, all specimens underwent heat treatment to relieve internal residual stress, followed by sandblasting and polishing according to the standardized protocol described in our previous study^17^.

### Construction of the in-vitro experimental model

**Fig. 2:**
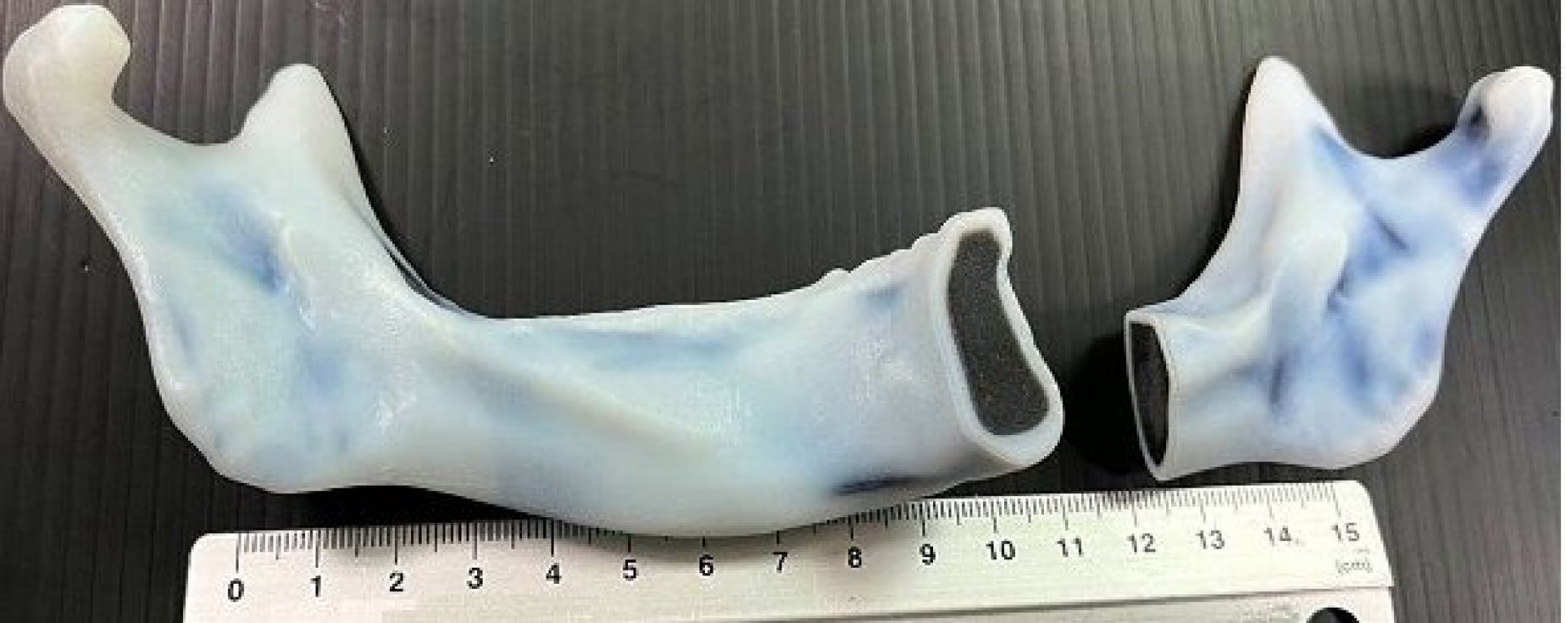
The mandibular model.

The three-dimensional mandible model used in this study was also derived from the same patient’s CBCT data to ensure anatomical fidelity. To realistically replicate the heterogeneous mechanical behavior of native bone, the model was designed as a composite structure consisting of two materials with distinct mechanical properties, representing the outer cortical bone and the inner cancellous bone (Fig. 2).

**Fig. 3:**
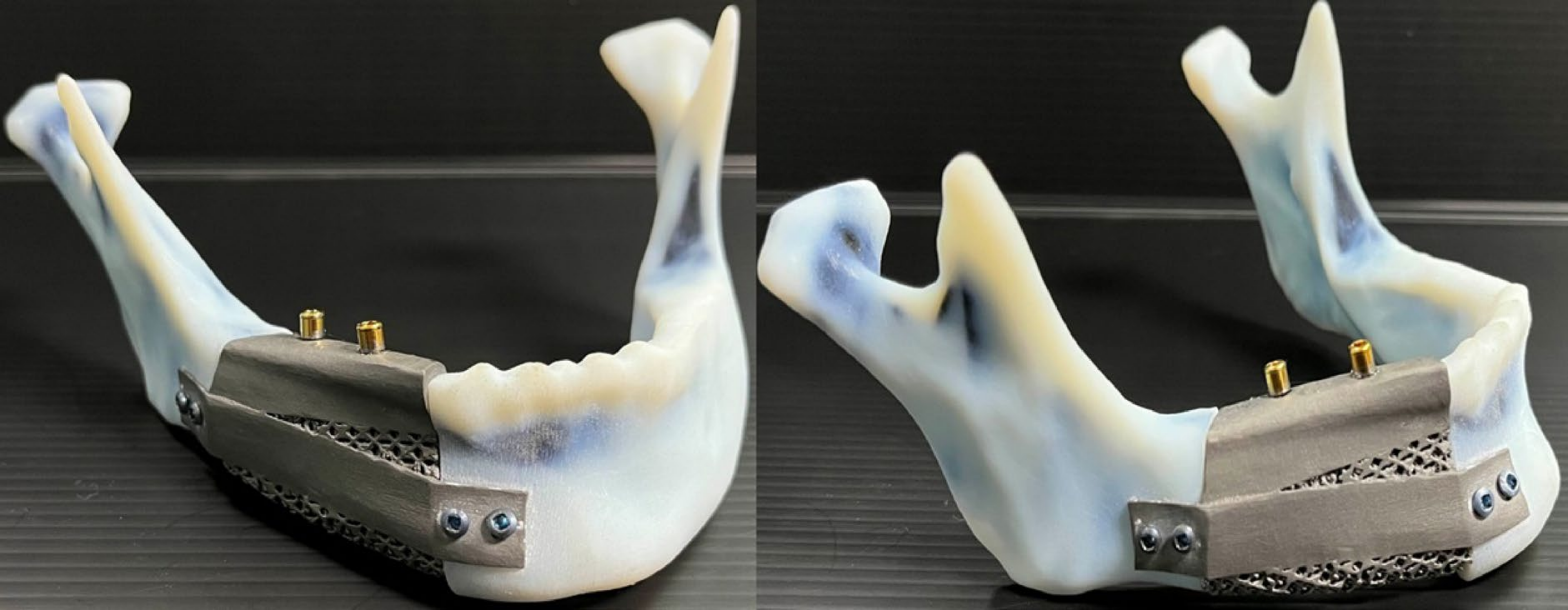
The assembled in-vitro experimental model.

The mandible model was fabricated using a Stratasys J850 PolyJet 3D printer (Stratasys, Eden Prairie, MN, USA). The cortical bone component was printed using the rigid material VeroWhitePlus (Stratasys), which exhibits an elastic modulus of 2–3 GPa, a tensile strength of 50–65 MPa, and a flexural modulus of 2.2–3.2 GPa. The cancellous bone component was fabricated from the rubber-like composite material Digital Material FLX9750-DM (Stratasys), characterized by a Shore hardness of 45–50, a tensile strength of 1.9–3.0 MPa, and an elongation at break of 95–110%. Subsequently, the implant was secured to the mandible model using four fixation screws (length: 12 mm; head diameter: 4 mm; neck diameter: 2.7 mm; thread pitch: 1.26 mm) as shown in Fig. 3.

### Loading conditions and strain measurement

**Fig. 4:**
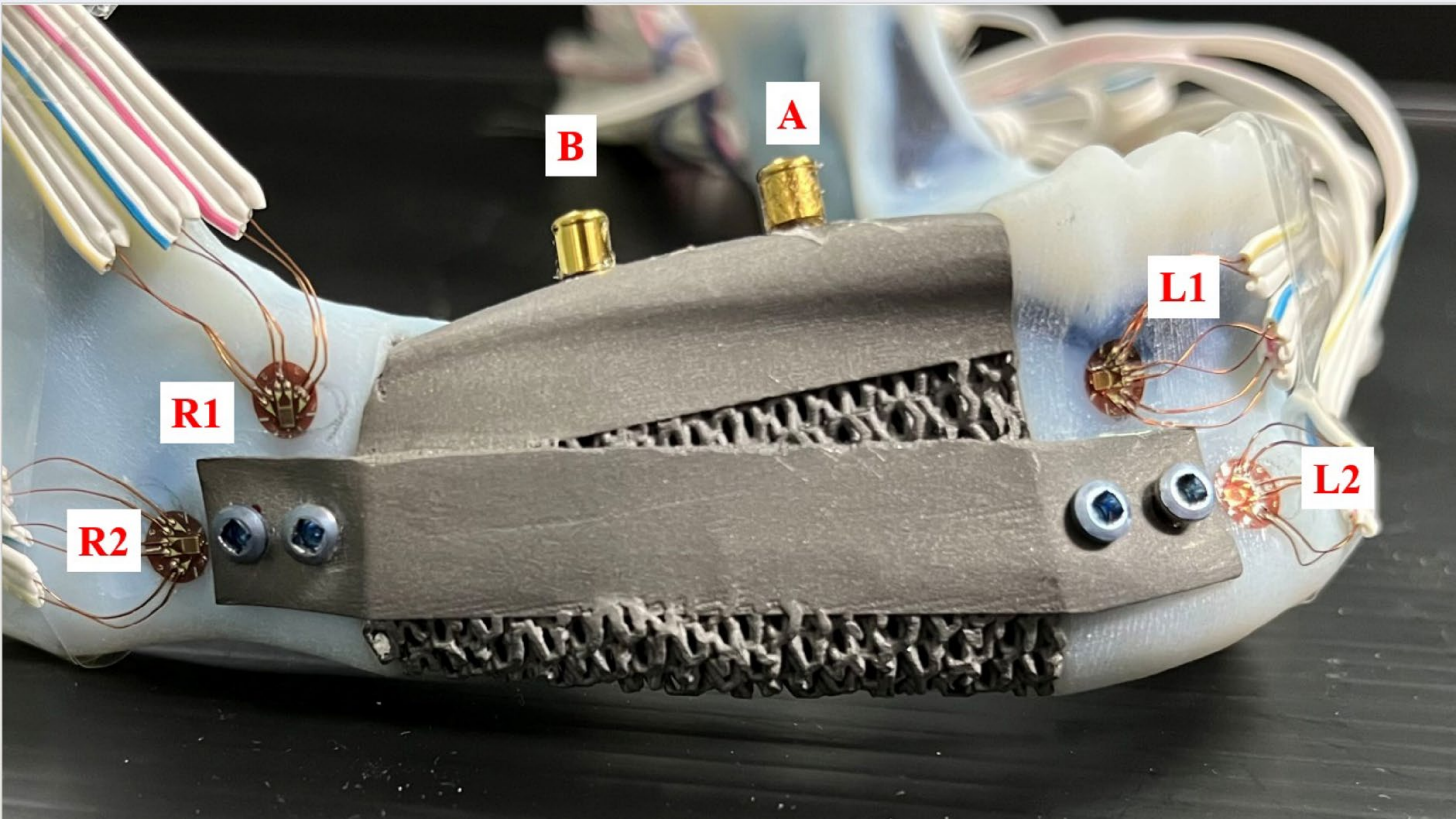
Locations of the strain gauges and the load application points.

To evaluate the surface strain distribution on the bone model surrounding the implant, triaxial strain gauges (Kyowa Electronic Instruments Co., Ltd., Tokyo, Japan) were bonded to four critical locations on the mandibular model (Fig. 4). Prior to gauge attachment, the model surface was gently abraded using 600-grit sandpaper and cleaned with alcohol to ensure optimal adhesion. The strain gauges were affixed using a cyanoacrylate-based adhesive recommended for precision strain measurement. All gauges were connected to a data acquisition (DAQ) system (CompactDAQ, National Instruments, Austin, TX, USA) integrated with control software (LabVIEW SignalExpress 3.0, National Instruments, Austin, TX, USA) to continuously record microstrain (µε) variations at each measurement site in real time.

After measuring the µε at the four measurement sites (L1, L2, R1, and R2), the maximum (ε_max) and minimum (ε_min) principal strains were calculated using the following equations:

1 $$\varepsilon _{{{\mathrm{max}}}} = {\text{ 1}}/{\mathrm{2}}\left( {\varepsilon _{{\mathrm{a}}} + \varepsilon _{{\mathrm{c}}} } \right) + {\mathrm{1}}/{\mathrm{2}}\sqrt {\left[ {\left( {\varepsilon _{{\mathrm{a}}} {-}\varepsilon _{{\mathrm{c}}} } \right)^{{\mathrm{2}}} + \left( {{\mathrm{2}}\varepsilon _{{\mathrm{b}}} {-}\varepsilon _{{\mathrm{a}}} {-}\varepsilon _{{\mathrm{c}}} } \right)^{{\mathrm{2}}} } \right]}$$

2 $$\varepsilon _{{{\mathrm{min}}}} = {\text{ 1}}/{\mathrm{2}}\left( {\varepsilon _{{\mathrm{a}}} + \varepsilon _{{\mathrm{c}}} } \right) - {\mathrm{1}}/{\mathrm{2}}\sqrt {\left[ {\left( {\varepsilon _{{\mathrm{a}}} - \varepsilon _{{\mathrm{c}}} } \right)^{{\mathrm{2}}} + \left( {{\mathrm{2}}\varepsilon _{{\mathrm{b}}} - \varepsilon _{{\mathrm{a}}} - \varepsilon _{{\mathrm{c}}} } \right)^{{\mathrm{2}}} } \right]}$$

**Fig. 5:**
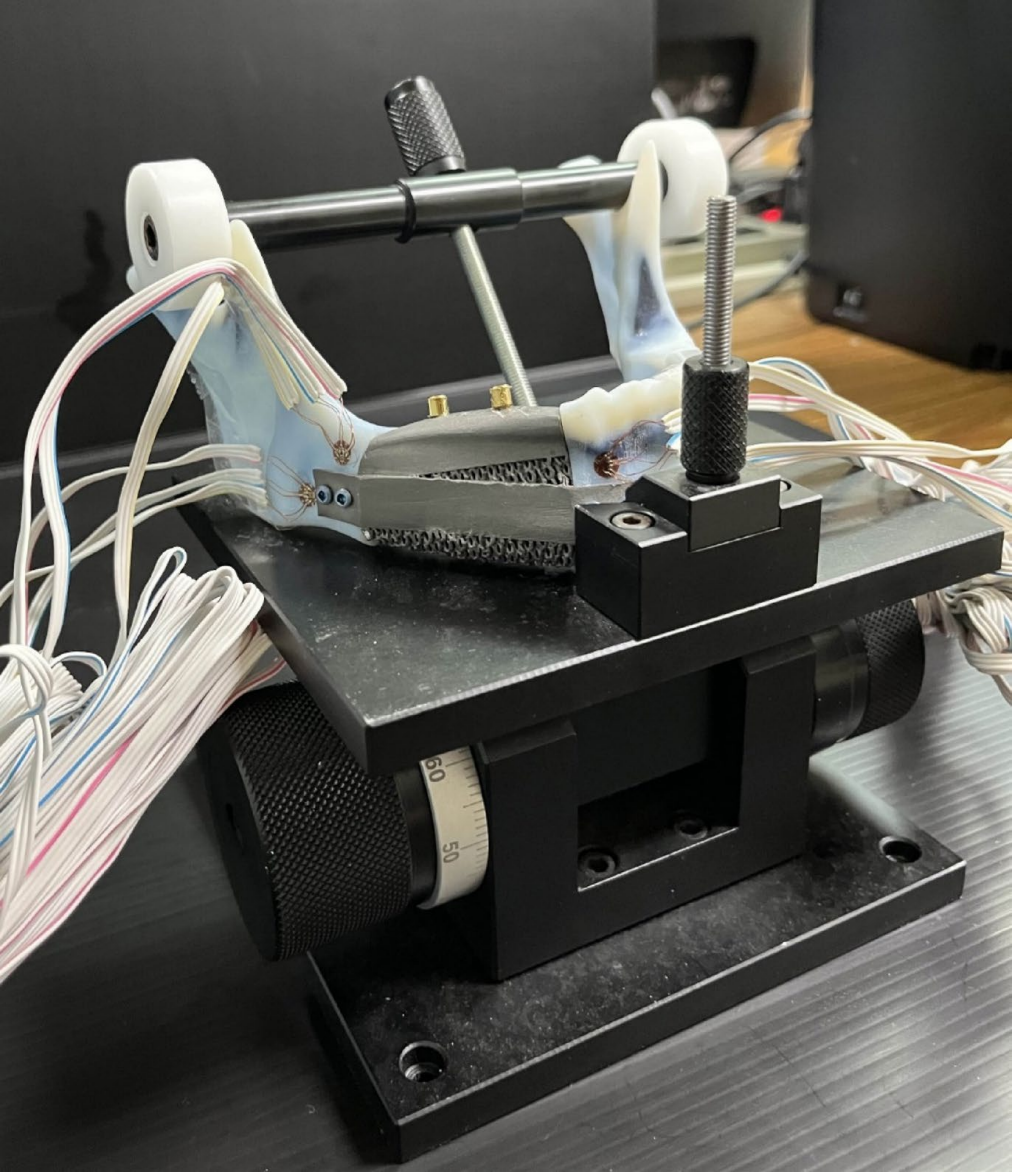
The custom-designed fixture for the in-vitro experimental model.

**Fig. 6:**
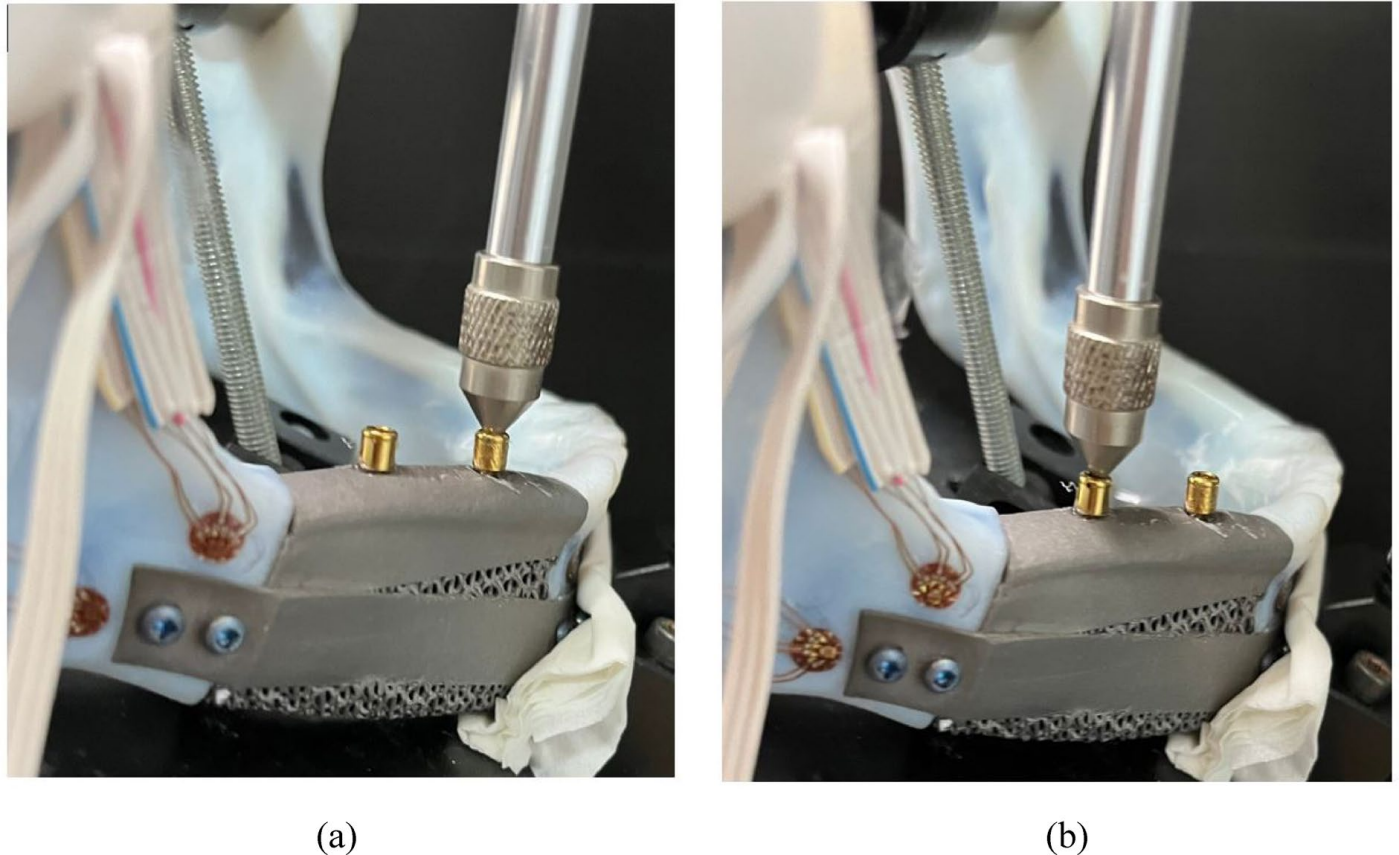
The experimental setup for loading at **a** Point A, and **b** Point B.

For the in-vitro loading experiment, the complete mandible–implant assembly was fixed onto a custom-designed jig (Fig. 5) and tested using a universal testing machine (JSV-H1000, Japan Instrumentation System, Nara, Japan). The load was applied at a constant crosshead displacement rate of 1 mm/min until the target load of 100 N was reached. For each implant configuration, the loading tests were performed sequentially at Point A and then at Point B (Fig. 6). To ensure data reliability, each loading condition was repeated for seven independent trials (*n* = 7), and the corresponding strain data were recorded for subsequent analysis.

The statistical results of all measurements, including the mean and standard deviation (SD) of principal strains were recorded and analyzed.

### Statistical analysis

Statistical analysis was performed using SPSS software (IBM Corp., Armonk, NY, USA). Each experimental condition was evaluated with seven independent repetitions (*n* = 7). The normality of the data distribution was first assessed using the Shapiro–Wilk test. To evaluate the effects of different implant designs and loading conditions on the resulting principal strains, comparative analyses were conducted as follows: When the data met the assumption of normality, a one-way analysis of variance (ANOVA) was utilized, followed by Bonferroni and Tukey’s post-hoc tests for multiple comparisons. Conversely, when the data were not normally distributed, the Kruskal–Wallis test was applied, followed by the Dunn–Bonferroni post-hoc method. All statistical tests were two-tailed, and the level of statistical significance was set at *p* < 0.05 (α = 0.05).

## Data Availability

The data that support the findings of this study are available from the corresponding author upon reasonable request.
